# Multi-Target Tracking Based on Multi-Bernoulli Filter with Amplitude for Unknown Clutter Rate

**DOI:** 10.3390/s151229804

**Published:** 2015-12-04

**Authors:** Changshun Yuan, Jun Wang, Peng Lei, Yanxian Bi, Zhongsheng Sun

**Affiliations:** School of Electronic and Information Engineering, Beihang University, Beijing 100191, China; yuanchang61@buaa.edu.cn (C.Y.); peng.lei@buaa.edu.cn (P.L.); biyanxian@buaa.edu.cn (Y.B.); sunzhsh@buaa.edu.cn (Z.S.)

**Keywords:** multi-Bernoulli filter, random finite set, multi-target tracking, amplitude information, clutter rate estimation, sequential Monte-Carlo

## Abstract

Knowledge of the clutter rate is of critical importance in multi-target Bayesian tracking. However, estimating the clutter rate is a difficult problem in practice. In this paper, an improved multi-Bernoulli filter based on random finite sets for multi-target Bayesian tracking accommodating non-linear dynamic and measurement models, as well as unknown clutter rate, is proposed for radar sensors. The proposed filter incorporates the amplitude information into the state and measurement spaces to improve discrimination between actual targets and clutters, while adaptively generating the new-born object random finite sets using the measurements to eliminate reliance on prior random finite sets. A sequential Monte-Carlo implementation of the proposed filter is presented, and simulations are used to demonstrate the proposed filter’s improvements in estimation accuracy of the target number and corresponding multi-target states, as well as the clutter rate.

## 1. Introduction

As a system, the radar has been widely applied in both civil and military areas due to its all-weather, day and night capability compared with optical and infrared sensors [1]. One of its most significant and important applications is target tracking. With the advancement of radar systems, target tracking has focused on multi-target tracking. The objective of multi-target tracking is to jointly estimate the unknown and time-varying number of targets and the corresponding states of multiple targets from measurements. Since measurements produced by radars usually include dense clutters, multi-target tracking becomes a challenging problem [2,3,4]. In many studies on the topic of multi-target tracking, it is usually assumed that the clutter rate is known and time-invariant as *a priori* parameter. However, in real-world applications, this parameter is often previously unknown and its value may be time-varying as the environment changes. Therefore, the ability of tracking multiple targets with an unknown clutter rate is very important in practice.

To solve this problem, some approaches for traditional multi-target tracking with unknown clutter rate were proposed, such as the non-parametric Joint Probabilistic Data Association (JPDA) [5] and the Joint Integrated Probabilistic Data Association (JIPDA) [6]. However, due to the necessary process of associations between appropriate targets and measurements, the computational load of the aforementioned techniques increases exponentially as the number of targets and measurements increases. This indicates that they may be unsuitable in many situations, *i.e.*, ground moving targets tracking.

Mahler recently proposed a random finite set (RFS) approach, which provided an elegant and mathematical Bayesian formulation to the multi-target tracking problem [4] and which could avoid associations. In particular, the development of the probability hypothesis density (PHD) [7] and the cardinalized probability hypothesis density (CPHD) [8] filters, including sequential Monte-Carlo (SMC) and Gaussian mixture (GM) implementations [9,10,11], as well as convergence analysis [12,13], have verified the practicality of the RFS approach. Furthermore, multi-target tracking with unknown clutter rate has been addressed with methods based on PHD and CPHD filters [14,15]. A closed form GM implementation for linear scenarios is proposed in [15]. Although it is possible to extend this work to general non-linear scenarios via SMC implementations, this solution may not achieve acceptable performance due to the necessary clustering process, which has the drawback of being inherently unreliable in state estimations.

A significant step towards addressing this problem was the multi-Bernoulli filter (MBerF), which was itself based on RFS; it proposed by Mahler [4] and modified by Vo [16]. Recently, the MBerF has been widely applied to audio and video tracking [17,18,19], sensor network tracking [20], and cell tracking [21]. Compared with the SMC-PHD and SMC-CPHD filter, the key advantage of the SMC implementation of MBerF is to avoid the additional clustering process, and achieve a more efficient and reliable state estimation of multiple targets. Moreover, Vo proposed a novel MBerF with unknown clutter rate (UCR-MBerF) which was inspired by the method used for the PHD/CPHD filters in [22]. However, this filter produced a large variance in the clutter rate and cardinality estimation and a bias in both of them, which became noticeable as the clutter rate increased. Moreover, it was assumed that the new-born object RFSs were known *a priori*. However, prior knowledge of new-born object RFSs could not be achieved in practice.

In this paper, we propose an improved UCR-MBerF, which incorporates amplitude information in the state and measurement spaces. The proposed filter is abbreviated as UCR-MBerF-AI. Our major innovations are given below:

- We derive novel prediction and update equations which augment the state and measurement spaces with the amplitude information. In radar sensors, measurements not only include the object’s position, but also contain information about the signal’s amplitude. The signal amplitude from an actual target is typically stronger than that of clutter. Therefore, it provides valuable information, which is useful to determine whether the measurement is from an actual target or from clutter.
- We adaptively generate the new-born object RFSs using the measurements so as to eliminate reliance of the prior new-born object RFSs.
- We carry out the SMC implementation of the proposed filter for general non-linear multi-target tracking scenarios.

The structure of this paper is as follows: a RFS model with amplitude for radar sensors with unknown clutter rate is introduced in Section 2. Section 3 presents an improved MBerF without the need of the prior clutter rate and new-born object RFSs assumptions, as well as a generic SMC implementation of the proposed filter for non-linear multi-target scenarios. Section 4 shows the numerical simulations with a non-linear multi-target scenario. Conclusions are finally given in Section 5.

## 2. RFS Model with Amplitude Information for Radar Sensors under Unknown Clutter Rate

In this section, we show how amplitude information is incorporated into an RFS model that accommodates unknown clutter rate scenarios. At the same time, we present the likelihood functions for the clutter and actual target by adopting radar amplitude models.

### 2.1. RFS Model with Amplitude Information for Unknown Clutter Rate

Suppose that there are N(k) actual targets with states $x_{k,1}\ldots ,x_{k,N(k)}$ and M(k) observations with measurements $z_{k,1}\ldots ,z_{k,M(k)}$ at time k. The multi-target states and observations are then represented by $X_{k}=\{x_{k,1}\ldots ,x_{k,N(k)}\}$ and $Z_{k}=\{z_{k,1}\ldots ,z_{k,M(k)}\}$, respectively [4]. In this paper, we consider an augmented state which includes kinematic $\tilde{x}$, parameter A of amplitude information and label u for actual targets and clutters, *i.e.*, $x=\left(\tilde{x},A,u\right)$, and each measurement contains position $\tilde{z}$ and amplitude r of the return signal, *i.e.*, $z=\left(\tilde{z},r\right)$.

Given a multi-target state $X_{k-1}$ at time k−1, state $X_{k}$ is represented as the union of surviving objects that survive from $X_{k-1}$ with the survival probability $p_{s,k}\left(x\right)$, and new-born objects that appear at time k. In order to distinguish new-born objects from the surviving objects, the label β is used, *i.e.*, $X_{k}=\underset{\beta =0,1}{\cup }X_{k|k-1,\beta }$. Given $X_{k}$, the measurements $Z_{k}$ are written as $Z_{k}=\left(\underset{x\in X_{k}}{\cup }\sum \left(x\right)\right)$, where ∑(x) is the RFS which has a probability $p_{D,k}\left(x\right)g_{k}\left(z|x\right)$ of containing a measurement {z} or is empty with probability $1-p_{D,k}\left(x\right)$ [4]. Note that the objects contain clutters and actual targets.

In the following, it is assumed that clutters and actual targets are statistically independent, while arbitrary functions defined on the state space will be denoted by $f\left(\tilde{x},A,u\right)=f_{u}\left(\tilde{x},A\right)$. The convention that u=1 denotes actual targets and u=0 denotes clutters as well as β=1 denotes surviving objects and β=0 denotes new-born objects will be adopted throughout this paper.

### 2.2. Amplitude Likelihoods for Radar Sensors

Let us assume that the amplitude of the return signal is independent of the object’s kinematic state. Then, the likelihood $g_{k}\left(z|x\right)$ is given by:

(1) $$g_{k}\left(z|x\right)=g_{u,k}\left(\tilde{z}|\tilde{x}\right)g_{u,k}\left(r|A\right)$$

In this paper, we adopt the Rice amplitude model, in which the probability densities of the amplitude of clutter and actual target before threshold detector are represented as in [1]:

(2) $$\left\{ \begin{array}{l} g_{0,k}\left(r|A\right)=\frac{r}{\psi^{2}}\exp\left(-\frac{r^{2}}{2\psi^{2}}\right)\;r\geq 0\\ g_{1,k}\left(r|A\right)=\frac{r}{\psi^{2}}I_{0}\left(\frac{rA}{\psi^{2}}\right)\exp\left(-\frac{r^{2}+A^{2}}{2\psi^{2}}\right)\;r\geq 0 \end{array}\right.$$

where $I_{0}\left(\cdot \right)$ is the modified Bessel function, ψ is the standard deviation of the noise.

**Figure 1:**
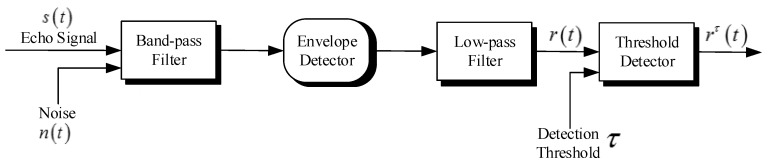
Flow diagram of signal processing for a typical radar receiver.

In the radar system, a typical detection process is to find local maxima among all measurements followed by thresholding at a certain level τ [1]. The flow diagram of signal processing sequence for a radar receiver is shown in Figure 1.

If the amplitude likelihood functions for the measurements which exceed the detection threshold are denoted as $g_{0,k}^{\tau }\left(r|A\right)$ and $g_{1,k}^{\tau }\left(r|A\right)$, then we have:

(3) $$\left\{ \begin{array}{l} g_{0,k}^{\tau }\left(r|A\right)=\frac{g_{0,k}\left(r|A\right)}{p_{FA,k}^{\tau }}\\ g_{1,k}^{\tau }\left(r|A\right)=\frac{g_{1,k}\left(r|A\right)}{p_{D,k}^{\tau }} \end{array}\right.$$

where:

(4) $$\left\{ \begin{array}{c} p_{FA,k}^{\tau }=\int_{\tau }^{\infty }g_{0,k}\left(r|A\right)dr=\exp\left(-\frac{\tau^{2}}{2\psi^{2}}\right)\\ p_{D,k}^{\tau }=\int_{\tau }^{\infty }g_{1,k}\left(r|A\right)dr=Q\left[\sqrt{\frac{A^{2}}{\psi^{2}}},\sqrt{2\ln\left(\frac{1}{p_{FA,k}^{\tau }}\right)}\right] \end{array}\right.$$

are the probabilities of false alarm and detection. $Q\left[\alpha ,\beta \right]=\int_{\beta }^{\infty }\zeta I_{0}\left(\alpha \zeta \right)\exp\left(-\left(\alpha^{2}+\zeta^{2}\right)/2\right)d\zeta$ is the MarcumQ function and can be computed by numerical integration offline.

## 3. UCR-MBerF-AI and SMC Implementation

In this section, we derive an improved UCR-MBerF by incorporating the amplitude information into a single object state and the measurement and adaptively generate the new-born object RFSs. In Section 3.1, we describe the UCR-MBerF-AI, while further on, in Section 3.2, we present a detailed SMC implementation of the proposed UCR-MBerF-AI.

### 3.1. UCR-MBerF-AI

The UCR-MBerF-AI can be derived from the UCR-MBerF with a particular chosen state and measurement space. This approach is described below. First, analogous to the UCR-MBerF, an unknown clutter rate is accommodated by modelling individual clutters which have their own separate models for transitions, detections and likelihoods, as well as formation and dissolution. Second, we incorporate amplitude information into the state and measurement variables and generate the augmented variables which were introduced in Section 2.1. However, the UCR-MBerF assumes that the new-born object RFSs are known *a priori*, which cannot be achieved in practice. To solve this problem, we propose a method inspired by [23], namely the formulation of the new-born object RFSs using measurement information. The UCR-MBerF-AI is fundamentally different to the approach proposed in [23], so it solves problems that are not possible to solve with the latter. The prediction and update step of the UCR-MBerF-AI are shown as follows:

#### 3.1.1. UCR-MBerF-AI Prediction 

The posterior multi-target density (Po-MTD) for MBerF is approximated by a finite and time-varying number of Bernoulli RFSs ${\left\{\left(r_{k}^{\left(i\right)},p_{k}^{\left(i\right)}\right)\right\}}_{i=1}^{M_{k}}$ where $r_{k}^{\left(i\right)}$ and $p_{k}^{\left(i\right)}$ denote the existence probability and the state probability density for the *i*^th^ Bernoulli components, respectively [16]. Then, at time k−1, the Po-MTD can be written as $\pi_{k-1}={\left\{\left(r_{k-1}^{\left(i\right)},p_{u,k-1}^{\left(i\right)}\right)\right\}}_{i=1}^{M_{k-1}}$.

As described in Section 2.1, the predicted objects include the new-born objects and surviving objects. Then, the predicted multi-target density (Pr-MTD) is given by the union of surviving and new-born Bernoulli components [22] and is calculated as follows:

(5) $$\pi_{k|k-1}=\underset{\beta =0,1}{\cup }{\left\{\left(r_{k|k-1,\beta }^{\left(i\right)},p_{u,k|k-1,\beta }^{\left(i\right)}\right)\right\}}_{i=1}^{M_{k|k-1,\beta }}\;$$

where:

$$r_{k|k-1,\beta =1}^{\left(i\right)}=r_{k-1}^{\left(i\right)}\sum_{u=0,1}\langle p_{u,k-1}^{\left(i\right)},p_{S,u,k}\rangle$$

$$p_{u,k|k-1,\beta =1}^{\left(i\right)}\left(\tilde{x},A\right)=\frac{\langle f_{u,k|k-1}\left(\tilde{x},A|\cdot ,\cdot \right),p_{u,k-1}^{\left(i\right)}p_{S,u,k}\rangle }{\langle p_{u,k-1}^{\left(i\right)},p_{S,u,k}\rangle }$$

${\left\{\left(r_{k|k-1,\beta =0}^{\left(i\right)},p_{u,k|k-1,\beta =0}^{\left(i\right)}\right)\right\}}_{i=1}^{M_{k|k-1,\beta =0}}$ are the new-born object RFSs, 〈·,·〉 denotes an inner product $f_{u,k|k-1}\left(\tilde{x},A|\tilde{\zeta },\varsigma \right)$ is the single object transition density given a previous state $\left(\tilde{\zeta },\varsigma \right)$, while $p_{S,u,k}(\tilde{\zeta },\varsigma )$ is an object’s probability of survival given a previous state $\left(\tilde{\zeta },\varsigma \right)$.

#### 3.1.2. UCR-MBerF-AI Update

At time k, the new-born objects are driven by measurements and are always detected, so the probability of detection is always 1. According to the analysis in Section 2.2, for surviving clutter and actual targets, the probability of detection only depends on the threshold τ and parameter A of amplitude information, then the probability of detection is given by:

(6) $$p_{D,u,k,\beta =1}\;(\tilde{x},A)=\left\{ \begin{array}{l} \;p_{D,k}^{\tau }\;u=1\\ \;p_{FA,k}^{\tau }\;u=0 \end{array}\right.$$

If the Pr-MTD is calculated in the predict step as shown in Equation (5), then for a set of receiving measurements $Z_{k}$, the Po-MTD is written as a union of legacy and updated components for the new-born and surviving objects, respectively [22]. However, since the probability of detection is always 1 for new-born objects, the legacy components for new-born objects can be disregarded. Consequently, the final form can be written as:

(7) $$\pi_{k}\;={\left\{\left(r_{L,k}^{\left(i\right)},p_{L,u,k}^{\left(i\right)}\right)\right\}}_{i=1}^{M_{k}^{L}}\;{\cup \left\{\left(r_{U,k}\left(z\right),p_{U,u,k}\left(\cdot ;z\right)\right)\right\}}_{z\in Z_{k}}$$

where:

$$M_{k}^{L}=M_{k|k-1,\beta =1}$$

$$r_{L,k}^{\left(i\right)}=\sum_{u=0,1}r_{L,u,k}^{\left(i\right)}$$

$$r_{L,u,k}^{\left(i\right)}=\frac{r_{k|k-1,\beta =1}^{\left(i\right)}\langle p_{u,k|k-1,\beta =1}^{\left(i\right)},1-p_{D,u,k,\beta =1}\rangle }{1-r_{k|k-1,\beta =1}^{\left(i\right)}\sum_{u^{\prime }=0,1}\langle p_{u,k|k-1,\beta =1}^{\left(i\right)},1-p_{D,u^{\prime },k,\beta =1}\rangle }$$

$$p_{L,u,k}^{\left(i\right)}\left(\tilde{x},A\right)=\frac{\left(1-p_{D,u,k,\beta =1}\;(\tilde{x},A)\right)p_{u,k|k-1,\beta =1}^{\left(i\right)}\left(\tilde{x},A\right)}{\sum_{u^{\prime }=0,1}\langle p_{u^{\prime },k|k-1,\beta =1}^{\left(i\right)},1-p_{D,u^{\prime },k,\beta =1}\;\rangle }$$

$$r_{U,k}\left(z\right)=\sum_{u=0,1}r_{U,u,k}\left(z\right)$$

$$r_{U,u,k}\left(z\right)\;=\frac{\sum_{\beta =0,1}\sum_{i=1}^{M_{k|k-1,\beta }}\frac{r_{k|k-1,\beta }^{\left(i\right)}\left(1-r_{k|k-1,\beta }^{\left(i\right)}\right)\langle p_{u,k|k-1,\beta }^{\left(i\right)},g_{u,k}\left(\tilde{z}|\tilde{x}\right)g_{u,k}^{\tau }\left(r|A\right)p_{D,u,k,\beta }\rangle }{{\left(1-r_{k|k-1,\beta }^{\left(i\right)}\sum_{u^{\prime }=0,1}\langle p_{u^{\prime },k|k-1,\beta }^{\left(i\right)},p_{D,u^{\prime },k,\beta }\rangle \right)}^{2}}}{\sum_{\beta =0,1}\sum_{i=1}^{M_{k|k-1,\beta }}\frac{r_{k|k-1,\beta }^{\left(i\right)}\sum_{u^{\prime }=0,1}\langle p_{u^{\prime },k|k-1,\beta }^{\left(i\right)},g_{u^{\prime },k}\left(\tilde{z}|\tilde{x}\right)g_{u^{\prime },k}^{\tau }\left(r|A\right)p_{D,u^{\prime },k,\beta }\rangle }{1-r_{k|k-1,\beta }^{\left(i\right)}\sum_{u^{\prime }=0,1}\langle p_{u^{\prime },k|k-1,\beta }^{\left(i\right)},p_{D,u^{\prime },k,\beta }\rangle }}$$

$$p_{U,u,k}\left(\tilde{x},A;z\right)=\frac{\sum_{\beta =0,1}\sum_{i=1}^{M_{k|k-1,\beta }}\frac{r_{k|k-1,\beta }^{\left(i\right)}}{\left(1-r_{k|k-1,\beta }^{\left(i\right)}\right)}p_{u,k|k-1,\beta }^{\left(i\right)}\left(\tilde{x},A\right)g_{u,k}\left(\tilde{z}|\tilde{x}\right)g_{u,k}^{\tau }\left(r|A\right)p_{D,u,k,\beta }\left(\tilde{x},A\right)}{\sum_{\beta =0,1}\sum_{u^{\prime }=0,1}\sum_{i=1}^{M_{k|k-1,\beta }}\frac{r_{k|k-1,\beta }^{\left(i\right)}}{\left(1-r_{k|k-1,\beta }^{\left(i\right)}\right)}\langle p_{u^{\prime },k|k-1,\beta }^{\left(i\right)},g_{u^{\prime },k}\left(\tilde{z}|\tilde{x}\right)g_{u^{\prime },k}^{\tau }\left(r|A\right)p_{D,u^{\prime },k,\beta }\rangle }\;$$

The amplitude likelihoods $g_{u,k}^{\tau }\left(r|A\right)$ are given by Equation (3).

Therefore, the UCR-MBerF-AI is different from the original UCR-MBerF in the sense that it not only considers the amplitude information as an augmented variable to improve discrimination between actual targets and clutters, but it also adopts a measurement-driven approach to adaptively generate the new-born object RFSs.

### 3.2. SMC Implementation

In this section, we present a SMC implementation of the proposed UCR-MBerF-AI. This can accommodate non-linear dynamic and measurement models. The convergence results for this SMC implementation still satisfy the convergence results for the conventional MBer filter in [24]. The SMC implementation is based on the sampling importance resampling (SIS) technique, where the transition density $f_{u,k|k-1}\left(\tilde{x},A|\tilde{\zeta },\varsigma \right)$ is used as the importance density. We assume the measurements include range and bearing. The prediction, update, resample, multi-target state and clutter rate estimation steps are given below:

#### 3.2.1. SMC Prediction

If at time k−1, the Po-MTD $\pi_{k-1}={\left\{\left(r_{k-1}^{\left(i\right)},p_{u,k-1}^{\left(i\right)}\right)\right\}}_{i=1}^{M_{k-1}}$ is given, and each $p_{u,k-1}^{\left(i\right)}$, $i=1,\ldots ,M_{k-1,}$, is written as $p_{u,k-1}^{\left(i\right)}(\tilde{x},A)=\sum_{j=1}^{L_{u,k-1}^{\left(i\right)}}w_{u,k-1}^{\left(i,j\right)}\delta_{\left({\tilde{x}}_{u,k-1}^{\left(i,j\right)},A_{u,k-1}^{\left(i,j\right)}\right)}(\tilde{x},A)$, then the surviving components of Equation (5) can be calculated as follows:

(8) $$r_{k|k-1,\beta =1}^{\left(i\right)}=r_{k-1}^{\left(i\right)}\sum_{u=0,1}\sum_{j=1}^{L_{u,k-1}^{\left(i\right)}}w_{u,k-1}^{\left(i,j\right)}p_{S,u,k}({\tilde{x}}_{u,k-1}^{\left(i,j\right)},A_{u,k-1}^{\left(i,j\right)})$$

(9) $$p_{u,k|k-1,\beta =1}^{\left(i\right)}\left(\tilde{x},A\right)=\sum_{j=1}^{L_{u,k-1}^{\left(i\right)}}{\tilde{w}}_{u,k|k-1,\beta =1}^{\left(i,j\right)}\delta_{\left({\tilde{x}}_{u,k|k-1,\beta =1}^{\left(i,j\right)},A_{u,k|k-1,\beta =1}^{\left(i,j\right)}\right)}(\tilde{x},A)$$

where:

(10) $${\tilde{w}}_{u,k|k-1,\beta =1}^{\left(i,j\right)}=\frac{w_{u,k-1}^{\left(i,j\right)}p_{S,u,k}({\tilde{x}}_{u,k-1}^{\left(i,j\right)},A_{u,k-1}^{\left(i,j\right)})}{\sum_{u^{\prime }=0,1}\sum_{j=1}^{L_{u^{\prime },k-1}^{\left(i\right)}}w_{u^{\prime },k-1}^{\left(i,j\right)}p_{S,u,k}({\tilde{x}}_{u^{\prime },k-1}^{\left(i,j\right)},A_{u^{\prime },k-1}^{\left(i,j\right)})}$$

$$\left({\tilde{x}}_{u,k|k-1,\beta =1}^{\left(i,j\right)},A_{u,k|k-1,\beta =1}^{\left(i,j\right)}\right)∼f_{u,k|k-1}\left(\cdot |{\tilde{x}}_{u,k-1}^{\left(i,j\right)},A_{u,k-1}^{\left(i,j\right)}\right)$$

Since the new-born objects given in Equation (5) could appear anywhere with equal probability in the state space, the new-born object RFSs must cover the entire state space. Although it is possible to cover the entire state space with the particles for the SMC-UCR-MBerF-AI, this approach is not possible in a practical scenario because a large number of particles would be necessary for the algorithm to work properly. Instead, we utilize the measurements to adaptively generate new-born object RFSs. We also find that the measurements near the predicted states ${\hat{X}}_{k|k-1}$ of estimated multi-target states ${\hat{X}}_{k-1}=\left\{{\hat{x}}_{k-1}^{\left(1\right)},{\hat{x}}_{k-1}^{\left(2\right)},\cdots ,{\hat{x}}_{k-1}^{\left({\hat{N}}_{k-1}\right)}\right\}$ are not likely to be obtained from the new-born object, so we remove measurements located near ${\hat{X}}_{k|k-1}$, and obtain the new measurement-driven set $Z_{\Gamma ,k}=\left\{z_{k}^{\left(1\right)},z_{k}^{\left(2\right)},\cdots ,z_{k}^{\left(\Gamma_{k}\right)}\right\}$.

Finally, the new-born components of Equation (5) can be calculated as follows:

(11) $$r_{k|k-1,\beta =0}^{\left(i\right)}=\sum_{u=0,1}r_{u,k,\beta =0}^{\left(i\right)}$$

(12) $$p_{u,k|k-1,\beta =0}^{\left(i\right)}\left(\tilde{x},A\right)=\sum_{j=1}^{N_{b}^{\left(i\right)}}{\tilde{w}}_{u,k|k-1,\beta =0}^{\left(i,j\right)}\delta_{\left({\tilde{x}}_{u,k|k-1,\beta =0}^{\left(i,j\right)},A_{u,k|k-1,\beta =0}^{\left(i,j\right)}\right)}(\tilde{x},A)$$

where

$$r_{u,k,\beta =0}^{\left(i\right)}=r_{u,\beta =0}^{\left(i\right)}=\frac{\nu_{k}^{b}}{V}$$

$${\tilde{w}}_{u,k|k-1,\beta =0}^{\left(i,j\right)}=\frac{1}{2N_{b}^{\left(i\right)}}$$

$$\left({\tilde{x}}_{u,k|k-1,\beta =0}^{\left(i,j\right)},A_{u,k|k-1,\beta =0}^{\left(i,j\right)}\right)∼b_{u,k}\left(\cdot |Z_{\Gamma ,k}\right)$$

The parameter $N_{b}^{\left(i\right)}$ is the number of particles per new-born object, $b_{u,k}\left(\cdot |Z_{\Gamma ,k}\right)$ is new-born object density given a set of measurements. The parameter $\nu_{k}^{b}$ is the expected number of new-born objects. The parameter V is the volume of the measurement space.

#### 3.2.2. SMC Update

If at time k, the Pr-MTD Equation (5) is given, and each $p_{u,k|k-1,\beta }^{\left(i\right)}$, $i=1,\ldots ,M_{k|k-1,\beta }$, is written as $p_{u,k|k-1,\beta }^{\left(i\right)}(\tilde{x},A)=\sum_{j=1}^{L_{u,k|k-1,\beta }^{\left(i\right)}}w_{u,k|k-1,\beta }^{\left(i,j\right)}\delta_{\left({\tilde{x}}_{u,k|k-1,\beta }^{\left(i,j\right)},A_{u,k|k-1,\beta }^{\left(i,j\right)}\right)}(\tilde{x},A)$, then the updated Po-MTD Equation (7) can be calculated as shown below:

(13) $$r_{L,k}^{\left(i\right)}=\sum_{u=0,1}r_{L,u,k}^{\left(i\right)}$$

(14) $$r_{L,u,k}^{\left(i\right)}=r_{k|k-1,\beta =1}^{\left(i\right)}\frac{1-\eta_{L,u,k,\beta =1}^{\left(i\right)}}{1-r_{k|k-1,\beta =1}^{\left(i\right)}\sum_{u^{\prime }=0,1}\eta_{L,u^{\prime },k,\beta =1}^{\left(i\right)}}$$

(15) $$p_{L,u,k}^{\left(i\right)}(\tilde{x},A)=\sum_{j=1}^{L_{u,k|k-1,\beta =1}^{\left(i\right)}}{\tilde{w}}_{L,u,k}^{\left(i,j\right)}\delta_{\left({\tilde{x}}_{u,k|k-1,\beta =1}^{\left(i,j\right)},A_{u,k|k-1,\beta =1}^{\left(i,j\right)}\right)}(\tilde{x},A)$$

(16) $$r_{U,k}\left(z\right)=\sum_{u=0,1}r_{U,u,k}\left(z\right)$$

(17) $$r_{U,u,k}\left(z\right)=\frac{\sum_{\beta =0,1}\sum_{i=1}^{M_{k|k-1,\beta }}\frac{r_{k|k-1,\beta }^{\left(i\right)}\left(1-r_{k|k-1,\beta }^{\left(i\right)}\right)\eta_{U,u,k,\beta }^{\left(i\right)}}{{\left(1-r_{k|k-1,\beta }^{\left(i\right)}\sum_{u^{\prime }=0,1}\eta_{L,u^{\prime },k,\beta }^{\left(i\right)}\right)}^{2}}}{\sum_{\beta =0,1}\sum_{i=1}^{M_{k|k-1,\beta }}\frac{r_{k|k-1,\beta }^{\left(i\right)}\sum_{u^{\prime }=0,1}\eta_{U,u^{\prime },k,\beta }^{\left(i\right)}}{1-r_{k|k-1,\beta }^{\left(i\right)}\sum_{u^{\prime }=0,1}\eta_{L,u^{\prime },k,\beta }^{\left(i\right)}}}$$

(18) $$p_{U,u,k}\left(\tilde{x},A;z\right)=\sum_{\beta =0,1}\sum_{i=1}^{M_{k|k-1,\beta }}\sum_{j=1}^{L_{u,k|k-1,\beta }^{\left(i\right)}}{\tilde{w}}_{U,u,k,\beta }^{\left(i,j\right)}\delta_{\left({\tilde{x}}_{u,k|k-1,\beta }^{\left(i,j\right)},A_{u,k|k-1,\beta }^{\left(i,j\right)}\right)}(\tilde{x},A)$$

where:

$$\eta_{L,u,k,\beta =1}^{\left(i\right)}=\sum_{j=1}^{L_{u,k|k-1,\beta =1}^{\left(i\right)}}w_{u,k|k-1,\beta =1}^{\left(i,j\right)}p_{D,u,k,\beta =1}({\tilde{x}}_{u,k|k-1,\beta =1}^{\left(i,j\right)},A_{u,k|k-1,\beta =1}^{\left(i,j\right)})$$

(19) $${\tilde{w}}_{L,u,k}^{\left(i,j\right)}=\frac{w_{u,k|k-1,\beta =1}^{\left(i,j\right)}(1-p_{D,u,k,\beta =1}({\tilde{x}}_{u,k|k-1,\beta =1}^{\left(i,j\right)},A_{u,k|k-1,\beta =1}^{\left(i,j\right)}))}{\sum_{u^{\prime }=0,1}\sum_{j=1}^{L_{u,k|k-1,\beta =1}^{\left(i\right)}}w_{u^{\prime },k|k-1,\beta =1}^{\left(i,j\right)}(1-p_{D,u^{\prime },k,\beta =1}({\tilde{x}}_{u^{\prime },k|k-1,\beta =1}^{\left(i,j\right)},A_{u^{\prime },k|k-1,\beta =1}^{\left(i,j\right)}))}$$

$$\eta_{L,u,k,\beta =0}^{\left(i\right)}=\sum_{j=1}^{L_{u,k|k-1,\beta =0}^{\left(i\right)}}w_{u,k|k-1,\beta =0}^{\left(i,j\right)}$$

$$\eta_{U,u,k,\beta =1}^{\left(i\right)}(z)=\sum_{j=1}^{L_{u,k|k-1,\beta =1}^{\left(i\right)}}w_{u,k|k-1,\beta =1}^{\left(i,j\right)}g_{u,k}\left(\tilde{z}|{\tilde{x}}_{u,k|k-1,\beta =1}^{\left(i,j\right)}\right)g_{u,k}^{\tau }\left(r|A_{u,k|k-1,\beta =1}^{\left(i,j\right)}\right)p_{D,u,k,\beta =1}({\tilde{x}}_{u,k|k-1,\beta =1}^{\left(i,j\right)},A_{u,k|k-1,\beta =1}^{\left(i,j\right)})$$

$$\eta_{U,u,k,\beta =0}^{\left(i\right)}(z)=\sum_{j=1}^{L_{u,k|k-1,\beta =0}^{\left(i\right)}}w_{u,k|k-1,\beta =0}^{\left(i,j\right)}\frac{1}{2\pi \sigma_{u,r}\sigma_{u,\theta }}g_{u,k}^{\tau }\left(r|A_{u,k|k-1,\beta =0}^{\left(i,j\right)}\right)$$

(20) $${\tilde{w}}_{U,u,k,\beta =1}^{\left(i,j\right)}=\frac{w_{u,k|k-1,\beta =1}^{\left(i,j\right)}\frac{r_{u,k|k-1,\beta =1}^{\left(i\right)}}{1-r_{u,k|k-1,\beta =1}^{\left(i\right)}}g_{u,k}\left(\tilde{z}|{\tilde{x}}_{u,k|k-1,\beta =1}^{\left(i,j\right)}\right)g_{u,k}^{\tau }\left(r|A_{u,k|k-1,\beta =1}^{\left(i,j\right)}\right)p_{D,u,k,\beta =1}({\tilde{x}}_{u,k|k-1,\beta =1}^{\left(i,j\right)},A_{u,k|k-1,\beta =1}^{\left(i,j\right)})}{\sum_{\beta =0,1}\sum_{u^{\prime }=0,1}\sum_{i=1}^{M_{k|k-1,\beta }}\frac{r_{u^{\prime },k|k-1,\beta }^{\left(i\right)}}{1-r_{u^{\prime },k|k-1,\beta }^{\left(i\right)}}\eta_{U,u^{\prime },k,\beta }^{\left(i\right)}(z)}$$

(21) $${\tilde{w}}_{U,u,k,\beta =0}^{\left(i,j\right)}=\frac{w_{u,k|k-1,\beta =0}^{\left(i,j\right)}\frac{r_{u,k|k-1,\beta =0}^{\left(i\right)}}{1-r_{u,k|k-1,\beta =0}^{\left(i\right)}}\frac{1}{2\pi \sigma_{u,r}\sigma_{u,\theta }}g_{u,k}^{\tau }\left(r|A_{u,k|k-1,\beta =1}^{\left(i,j\right)}\right)}{\sum_{\beta =0,1}\sum_{u^{\prime }=0,1}\sum_{i=1}^{M_{k|k-1,\beta }}\frac{r_{u^{\prime },k|k-1,\beta }^{\left(i\right)}}{1-r_{u^{\prime },k|k-1,\beta }^{\left(i\right)}}\eta_{U,u^{\prime },k,\beta }^{\left(i\right)}(z)}$$

Here, $\sigma_{u,r}$ and $\sigma_{u,\theta }$ are the standard deviations of the range and bearing noise, respectively. Note $g_{u,k}\left(\tilde{z}|{\tilde{x}}_{u,k|k-1,\beta =0}^{\left(i,j\right)}\right)=\frac{1}{2\pi \sigma_{u,r}\sigma_{u,\theta }}$ due to the measurement-driven new-born objects.

#### 3.2.3. Resampling

Similar to the UCR-MBerF [22], the number of particles for each Bernoulli component is re-allocated in scale to its existence probability, *i.e.*, $L_{k}^{\left(i\right)}=\max\left(r_{k}^{\left(i\right)}L_{\max},L_{\min}\right)$. In order to reduce the number of Bernoulli components, components with existence probability below a threshold G are discarded.

#### 3.2.4. Multi-Target State and Clutter Rate Estimation

Analogous to the UCR-MBerF [22], the estimated number of actual targets is ${\hat{N}}_{k}=\sum_{i=1}^{M_{k}}r_{1,k}^{\left(i\right)}$. The estimated individual actual target states ${\hat{X}}_{k}=\left\{{\hat{x}}_{k}^{\left(1\right)},{\hat{x}}_{k}^{\left(2\right)},\cdots ,{\hat{x}}_{k}^{\left({\hat{N}}_{k}\right)}\right\}$ are obtained from the means of the corresponding posterior density ${\hat{x}}_{k}^{\left(i\right)}=\sum_{j=1}^{L_{1,k}^{\left(i\right)}}w_{1,k}^{\left(i,j\right)}{\tilde{x}}_{1,k}^{\left(i,j\right)}$. The estimated clutter rate is ${\hat{\lambda }}_{c,k}=\sum_{i=1}^{M_{k}}r_{0,k}^{\left(i\right)}\sum_{j=1}^{L_{0,k}^{\left(i\right)}}w_{0,k}^{\left(i,j\right)}p_{D,0,k}^{\left(i,j\right)}$.

The processing steps of the proposed SMC-UCR-MBerF-AI are given in Algorithm 1.

**Algorithm 1.** Processing Steps of the SMC-UCR-MBerF-AI*Initialization*:
Let $\pi_{0}={\left\{\left(r_{0}^{\left(i\right)},p_{u,0}^{\left(i\right)}\right)\right\}}_{i=1}^{M_{0}}$ and $p_{u,0}^{\left(i\right)}(\tilde{x},A):={\left\{w_{u,0}^{\left(i,j\right)},\left({\tilde{x}}_{u,0}^{\left(i,j\right)},A_{u,0}^{\left(i,j\right)}\right)\right\}}_{j=1}^{L_{u,0}^{\left(i\right)}}$ represent the initial state.
*Input*:
Given $\pi_{k-1}={\left\{\left(r_{k-1}^{\left(i\right)},p_{u,k-1}^{\left(i\right)}\right)\right\}}_{i=1}^{M_{k-1}}$, $p_{u,k-1}^{\left(i\right)}(\tilde{x},A):={\left\{w_{u,k-1}^{\left(i,j\right)},\left({\tilde{x}}_{u,k-1}^{\left(i,j\right)},A_{u,k-1}^{\left(i,j\right)}\right)\right\}}_{j=1}^{L_{u,k-1}^{\left(i\right)}}$, the estimated multi-target states ${\hat{X}}_{k-1}$ and the current measurement set $Z_{k}$,
*Prediction*:
2.Compute the surviving Bernoulli components ${\left\{\left(r_{k|k-1,\beta =1}^{\left(i\right)},p_{u,k|k-1,\beta =1}^{\left(i\right)}\right)\right\}}_{i=1}^{M_{k|k-1,\beta =1}}\;$.
(a)Compute the existence probability $r_{k|k-1,\beta =1}^{\left(i\right)}$ using Equation (8), for $i=1,2,\cdots ,M_{k-1}$.(b)Compute the weight ${\tilde{w}}_{u,k|k-1,\beta =1}^{\left(i,j\right)}$ using Equation (10), for $j=1,2,\cdots ,L_{k-1}^{\left(i\right)}$, $i=1,2,\cdots ,M_{k-1}$ and u=0,1.(c)Draw the particle $\left({\tilde{x}}_{u,k|k-1,\beta =1}^{\left(i,j\right)},A_{u,k|k-1,\beta =1}^{\left(i,j\right)}\right)∼f_{u,k|k-1}\left(\cdot |{\tilde{x}}_{u,k-1}^{\left(i,j\right)},A_{u,k-1}^{\left(i,j\right)}\right)$, for $j=1,2,\cdots ,L_{k-1}^{\left(i\right)}$, for $i=1,2,\cdots ,M_{k-1}$ and u=0,1.3.Compute the new-born Bernoulli components ${\left\{\left(r_{k|k-1,\beta =0}^{\left(i\right)},p_{u,k|k-1,\beta =0}^{\left(i\right)}\right)\right\}}_{i=1}^{M_{k|k-1,\beta =0}}$.
(a)Remove measurements near the predicted states ${\hat{X}}_{k|k-1}$ of the estimated multi-target states ${\hat{X}}_{k-1}$ and obtain the rest of the measurements $Z_{\Gamma ,k}=\left\{z_{k}^{\left(1\right)},z_{k}^{\left(2\right)},\cdots ,z_{k}^{\left(\Gamma_{k}\right)}\right\}$, $M_{k|k-1,\beta =0}=\Gamma_{k}$.(b)Compute the existence probability $r_{k|k-1,\beta =0}^{\left(i\right)}=\sum_{u=0,1}r_{u,k,\beta =0}^{\left(i\right)}$, for $i=1,2,\cdots ,\Gamma_{k}$, and u=0,1.(c)Compute the weight ${\tilde{w}}_{u,k|k-1,\beta =0}^{\left(i,j\right)}=\frac{1}{2N_{b}^{\left(i\right)}}$ for $j=1,2,\cdots ,N_{b}^{\left(i\right)}$, for $i=1,2,\cdots ,\Gamma_{k}$ and u=0,1.(d)Draw the particle $\left({\tilde{x}}_{u,k|k-1,\beta =0}^{\left(i,j\right)},A_{u,k|k-1,\beta =0}^{\left(i,j\right)}\right)∼b_{u,k}\left(\cdot |Z_{\Gamma ,k}\right)$, for $j=1,2,\cdots ,N_{b}$, for $i=1,2,\cdots ,\Gamma_{k}$ and u=0,1.4.Using the union of the Bernoulli components, obtain the Pr-MTD as$\pi_{k|k-1}=\underset{\beta =0,1}{\cup }{\left\{\left(r_{k|k-1,\beta }^{\left(i\right)},p_{u,k|k-1,\beta }^{\left(i\right)}\right)\right\}}_{i=1}^{M_{k|k-1,\beta }}\;$
*Update*:
5.Compute the legacy Bernoulli components ${\left\{\left(r_{L,k}^{\left(i\right)},p_{L,u,k}^{\left(i\right)}\right)\right\}}_{i=1}^{M_{k}^{L}}$
(a)Compute the existence probability $r_{L,k}^{\left(i\right)}=\sum_{u=0,1}r_{L,u,k}^{\left(i\right)}$ via Equation (14), for $i=1,2,\cdots ,M_{k}^{L}$.(b)Compute the weight ${\tilde{w}}_{L,u,k|k}^{\left(i,j\right)}$ via Equation (19), for $j=1,2,\cdots ,L_{u,k|k-1,\beta =1}^{\left(i\right)}$, for $i=1,2,\cdots ,M_{k}^{L}$ and u=0,1.(c)Obtain the particle $\left({\tilde{x}}_{L,u,k|k}^{\left(i,j\right)},A_{L,u,k|k}^{\left(i,j\right)}\right)=\left({\tilde{x}}_{u,k|k-1,\beta =1}^{\left(i,j\right)},A_{u,k|k-1,\beta =1}^{\left(i,j\right)}\right)$, for $j=1,2,\cdots ,L_{u,k|k-1,\beta =1}^{\left(i\right)}$, for $i=1,2,\cdots ,M_{k}^{L}$ and u=0,1.6.Compute the updated Bernoulli components ${\left\{\left(r_{U,k}\left(z\right),p_{U,u,k}\left(\cdot ;z\right)\right)\right\}}_{z\in Z_{k}}$
(a)Compute the existence probability $r_{U,k}\left(z\right)=\sum_{u=0,1}r_{U,u,k}\left(z\right)$ via Equation (17), for $i=1,2,\cdots ,\left|Z_{k}\right|$.(b)Compute the weight ${\tilde{w}}_{U,u,k|k,\beta }^{\left(i,j\right)}$ via Equations (20) and (21), for $j=1,2,\cdots ,L_{u,k|k-1,\beta }^{\left(i\right)}$, for $i=1,2,\cdots ,M_{k|k-1,\beta }$, β=0,1 and u=0,1. Obtain the weight ${\tilde{w}}_{U,u,k|k,}^{\left(i,j\right)}=\underset{\beta =0,1}{\cup }{\tilde{w}}_{U,u,k|k,\beta }^{\left(i,j\right)}$.(c)Obtain the particle $\left({\tilde{x}}_{U,u,k|k}^{\left(i,j\right)},A_{U,u,k|k}^{\left(i,j\right)}\right)=\underset{\beta =0,1}{\cup }\left({\tilde{x}}_{u,k|k-1,\beta }^{\left(i,j\right)},A_{u,k|k-1,\beta }^{\left(i,j\right)}\right)$, for $j=1,2,\cdots ,L_{u,k|k-1,\beta }^{\left(i\right)}$, for $i=1,2,\cdots ,M_{k|k-1,\beta }$ and u=0,1.7.Using the union of the Bernoulli components, obtain the Po-MTD as$\pi_{k}\;={\left\{\left(r_{L,k}^{\left(i\right)},p_{L,u,k}^{\left(i\right)}\right)\right\}}_{i=1}^{M_{k}^{L}}\;{\cup \left\{\left(r_{U,k}\left(z\right),p_{U,u,k}\left(\cdot ;z\right)\right)\right\}}_{z\in Z_{k}}$
*Resample*:
8.Discard the Bernoulli components with existence probability below a threshold G and obtain $\pi_{k}={\left\{\left(r_{k}^{\left(i\right)},p_{u,k}^{\left(i\right)}\right)\right\}}_{i=1}^{M_{k}}$, and $p_{u,k}^{\left(i\right)}(\tilde{x},A):={\left\{{\tilde{w}}_{u,k|k}^{\left(i,j\right)},\left({\tilde{x}}_{u,k|k}^{\left(i,j\right)},A_{u,k|k}^{\left(i,j\right)}\right)\right\}}_{j=1}^{L_{u,k|k}^{\left(i\right)}}$.9.Resample $L_{k}^{\left(i\right)}=\max\left(r_{k}^{\left(i\right)}L_{\max},L_{\min}\right)$ times from ${\left\{{\tilde{w}}_{u,k|k}^{\left(i,j\right)},\left({\tilde{x}}_{u,k|k}^{\left(i,j\right)},A_{u,k|k}^{\left(i,j\right)}\right)\right\}}_{j=1}^{L_{u,k|k}^{\left(i\right)}}$ to obtain ${\left\{w_{u,k}^{\left(i,j\right)},\left({\tilde{x}}_{u,k}^{\left(i,j\right)},A_{u,k}^{\left(i,j\right)}\right)\right\}}_{j=1}^{L_{u,k}^{\left(i\right)}}$ with weights $w_{u,k}^{\left(i,j\right)}=1/L_{k}^{\left(i\right)}$ and $L_{u,k}^{\left(i\right)}=L_{k}^{\left(i\right)}$.
*Multi-target state and clutter rate estimation*:
10.Estimate number of actual targets ${\hat{N}}_{k}=\sum_{i=1}^{M_{k}}r_{1,k}^{\left(i\right)}$11.Estimate actual targets’ state ${\hat{X}}_{k}=\left\{{\hat{x}}_{k}^{\left(1\right)},{\hat{x}}_{k}^{\left(2\right)},\cdots ,{\hat{x}}_{k}^{\left({\hat{N}}_{k}\right)}\right\}$ with ${\hat{x}}_{k}^{\left(i\right)}=\sum_{j=1}^{L_{1,k}^{\left(i\right)}}w_{1,k}^{\left(i,j\right)}{\tilde{x}}_{1,k}^{\left(i,j\right)}$.12.Estimate clutter rate ${\hat{\lambda }}_{c,k}=\sum_{i=1}^{M_{k}}r_{0,k}^{\left(i\right)}\sum_{j=1}^{L_{0,k}^{\left(i\right)}}w_{0,k}^{\left(i,j\right)}p_{D,0,k}^{\left(i,j\right)}$.
*Output*:
$\pi_{k}={\left\{\left(r_{k}^{\left(i\right)},p_{u,k}^{\left(i\right)}\right)\right\}}_{i=1}^{M_{k}}$, $p_{u,k}^{\left(i\right)}(\tilde{x},A):={\left\{w_{u,k}^{\left(i,j\right)},\left({\tilde{x}}_{u,k}^{\left(i,j\right)},A_{u,k}^{\left(i,j\right)}\right)\right\}}_{j=1}^{L_{u,k}^{\left(i\right)}}$, ${\hat{X}}_{k}$, ${\hat{\lambda }}_{c,k}$

## 4. Simulation

In this section, we demonstrate the performance of the proposed UCR-MBerF-AI using a non-linear multi-target tracking scenario. We also compare it with the UCR-MBerF and the PHD filter for unknown clutter rate with amplitude information (UCR-PHDF-AI), whose new-born object RFSs are known *a priori*. The Optimal Subpattern Assignment (OSPA) metric [25] is used to evaluate the filters’ performance.

### 4.1. Simulation Scenario

**Figure 2:**
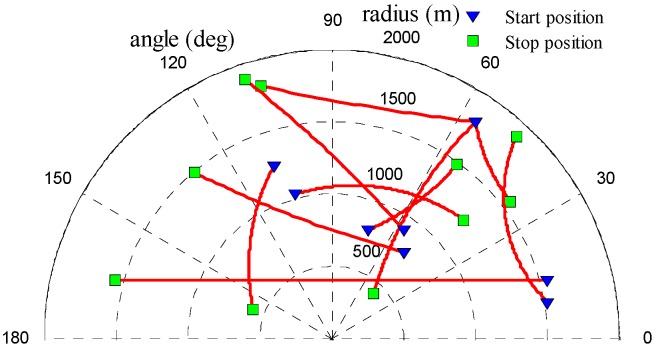
True target trajectories.

A non-linear multi-target scenario with the unknown clutter rate was used to demonstrate the performance of the UCR-MBerF-AI. The observation region is a half disc with a radius of 2000 m and has a total of ten targets. It is assumed that all of the targets have the same signal-to-noise ratio (SNR), defined in dB as $SNR=10\log\left(\frac{A^{2}}{2\psi^{2}}\right)$. Figure 2 shows the targets’ true trajectories, where the start and stop positions of each trajectory are indicated by the symbols ∇ and □, respectively.

#### 4.1.1. Actual Target Model

The actual target motion follows a coordinated turn model. The dynamics of an actual target’s state are written as:

(22) $$\left\{ \begin{array}{l} {\tilde{x}}_{k}=F_{1}(\omega_{k-1}){\tilde{x}}_{k-1}+G_{1}w_{k-1}\\ A_{k}=A_{k-1}+T\delta_{k-1} \end{array}\right.$$

where ${\tilde{x}}_{k}={\left[p_{x,k},{\dot{p}}_{x,k},p_{y,k},{\dot{p}}_{y,k},\omega_{k}\right]}^{T}$ contains the actual target’s position, velocity and turn rate, the transition matrix is $F_{1}(\omega )=\left[\begin{array}{ccccc} 1 & \frac{\sin\omega T}{\omega } & 0 & -\frac{1-\cos\omega T}{\omega } & 0\\ 0 & \cos\omega T & 0 & -\sin\omega T & 0\\ 0 & \frac{1-\cos\omega T}{\omega } & 1 & \frac{\sin\omega T}{\omega } & 0\\ 0 & \sin\omega T & 0 & \cos\omega T & 0\\ 0 & 0 & 0 & 0 & 1 \end{array}\right]$, process noise matrix is: $G_{1}=\left[\begin{array}{ccc} \frac{T^{2}}{2} & 0 & 0\\ T & 0 & 0\\ 0 & \frac{T^{2}}{2} & 0\\ 0 & T & 0\\ 0 & 0 & T \end{array}\right]$, the process noise distributions follow $w_{k-1}∼N\left(\cdot ;0,Q_{1}\right)$ $Q_{1}=diag\left(\left[\sigma_{1,w}^{2},\sigma_{1,w}^{2},\sigma_{1,\omega }^{2}\right]\right)$ and $\delta_{k-1}∼N\left(\cdot ;0,\sigma_{1,\delta }^{2}\right)$ with $\sigma_{1,w}=\text{5 m}/{\text{s}}^{\text{2}}$, $\sigma_{1,\omega }=\pi /180\;\text{rad}/\text{s}$ and $\sigma_{1,\delta }=3$, while the sampling time is T=1. Then, we have $f_{1,k|k-1}\left({\tilde{x}}_{k},A_{k}|{\tilde{x}}_{k-1},A_{k-1}\right)=N\left({\tilde{x}}_{k};{\tilde{x}}_{k-1},G_{1}Q_{1}{G_{1}}^{T}\right)N\left(A_{k};A_{k-1},\sigma_{1,\delta }^{2}\right)$. The survival probability of an actual target is fixed at $p_{S,1,k}=0.98$. The measurements of actual targets include bearing, range and amplitude with likelihoods given by:

(23) $$g_{1,k}\left({\tilde{z}}_{k}|{\tilde{x}}_{k}\right)=N\left({\tilde{z}}_{k};m_{\tilde{z},1,k}\left({\tilde{x}}_{k}\right),R_{\tilde{z},1,k}\right)$$

(24) $$g_{1,k}^{\tau }\left(r_{k}|A_{k}\right)=\frac{\frac{r_{k}}{\psi^{2}}I_{0}\left(\frac{r_{k}A_{k}}{\psi^{2}}\right)\exp\left(-\frac{{r_{k}}^{2}+{A_{k}}^{2}}{2\psi^{2}}\right)\;}{Q\left[\sqrt{\frac{{A_{k}}^{2}}{\psi^{2}}},\sqrt{2\ln\left(\frac{1}{p_{FA,k}^{\tau }}\right)}\right]}$$

where $m_{\tilde{z},1,k}\left(\tilde{x}\right)=\left[\arctan\left(p_{x,k}/p_{y,k}\right),\sqrt{p_{x,k}^{2}+p_{y,k}^{2}}\right]$ and $R_{\tilde{z},1,k}=diag\left({\left[\sigma_{1,\theta }^{2},\sigma_{1,r}^{2}\right]}^{T}\right)$ with $\sigma_{1,\theta }=\pi /180\text{ rad}$, $\sigma_{1,r}=5\;m$ and ψ=1. For every measurement $z_{k}^{\left(i\right)}\in Z_{\Gamma ,k}$, the new-born actual target particle position is given by [23]:

(25) $$\left\{ \begin{array}{l} p_{x,k}^{\left(i,j\right)}=p_{x,s}+\left(z_{k}^{\left(i\right)}\left[2\right]+\sigma_{1,r}\nu_{1}^{\left(j\right)}\right)\cos\left(z_{k}^{\left(i\right)}[1]+\sigma_{1,\theta }\nu_{2}^{\left(j\right)}\right)\\ p_{y,k}^{\left(i,j\right)}=p_{y,s}+\left(z_{k}^{\left(i\right)}\left[2\right]+\sigma_{1,r}\nu_{1}^{\left(j\right)}\right)\sin\left(z_{k}^{\left(i\right)}[1]+\sigma_{1,\theta }\nu_{2}^{\left(j\right)}\right) \end{array}\right.$$

where $j=1,\ldots ,N_{b}^{\left(i\right)}$, $z_{k}^{\left(i\right)}[1]$ and $z_{k}^{\left(i\right)}[2]$ are the bearing and range measurement respectively, and $\nu_{1}^{\left(n\right)},\nu_{2}^{\left(n\right)}∼N\left(\cdot ;0,1\right)$. $\left(p_{x,s},p_{y,s}\right)$ is the position of the sensor. The particle velocities follow ${\dot{p}}_{x,k}^{\left(i,j\right)},{\dot{p}}_{y,k}^{\left(i,j\right)}∼N\left(\cdot ;0,\sigma_{V}^{2}\right)$, where $\sigma_{V}=50\;m/s$, the particle turn rates are distributed as $\omega_{1,k}^{\left(i,j\right)},∼N\left(\cdot ;0,\sigma_{\omega }^{2}\right)$, with $\sigma_{\omega }=0.1\text{ rad}/s$, while the particle amplitudes follow $A_{k}^{\left(i,j\right)},∼N\left(\cdot ;m_{A},\sigma_{\delta }^{2}\right)$, where $\sigma_{\delta }=5$, $m_{A}=\sqrt{2\psi^{2}*10^{SNR/10}}$. The expected number of new-born actual targets at each scan is $\nu_{k}^{b}=0.32$.

#### 4.1.2. Clutter Model

The clutters are only modelled by their positions ${\tilde{x}}_{k}={\left[p_{x,k},p_{y,k}\right]}^{T}$, while their turn rates and velocities are ignored and parameter $A_{k}$ are set to zero. The positions of clutters follow a random walk model, with a transition density given by $f_{0,k|k-1}\left({\tilde{x}}_{k}|{\tilde{x}}_{k-1}\right)=N\left({\tilde{x}}_{k};{\tilde{x}}_{k-1},P_{x,0,k|k-1}\right)$, where $P_{x,0,k|k-1}=diag\left(\left[\sigma_{x}^{2},\sigma_{y}^{2}\right]\right)$ with $\sigma_{x}=1000\text{ m}$ and $\sigma_{y}=500\text{ m}$. The survival probability of clutters is fixed at $p_{S,0,k}=0.90$. The measurements of clutters are also include bearing, range and amplitude. Their corresponding likelihoods are given by:

(26) $$g_{0,k}\left({\tilde{z}}_{k}|{\tilde{x}}_{k}\right)=N\left({\tilde{z}}_{k};m_{\tilde{z},0,k}\left({\tilde{x}}_{k}\right),R_{\tilde{z},0,k}\right)$$

(27) $$g_{0,k}^{\tau }\left(r_{k}|A_{k}\right)=\frac{r_{k}}{\psi^{2}}\exp\left(\frac{\tau^{2}-{r_{k}}^{2}}{2\psi^{2}}\right)$$

where $m_{\tilde{z},0,k}\left(\tilde{x}\right)=\left[\arctan\left(p_{x,k}/p_{y,k}\right),\sqrt{p_{x,k}^{2}+p_{y,k}^{2}}\right]$ and $R_{\tilde{z},0,k}=diag\left({\left[\sigma_{0,\theta }^{2},\sigma_{0,r}^{2}\right]}^{T}\right)$ with $\sigma_{0,\theta }=20\pi /180\;rad$, $\sigma_{0,r}=400\;m$. The clutter birth process is similar that of the actual targets, but we only consider their position.

#### 4.1.3. Filter Parameters

In the proposed SMC implementation of the UCR-MBerF-AI, the maximum and minimum number of the particles per Bernoulli component are set to be $L_{\max}=1000$ and $L_{\min}=300$, respectively. The number of particles for each new-born object is $N_{b}^{\left(i\right)}=\max\left(r_{u,\beta =0}^{\left(i\right)}*L_{\max},L_{\min}\right)$. Additionally, $G=10^{-3}$ is the threshold of the probability of existence, while a maximum $T_{\max}=100$ is used for pruning the number of Bernoulli components.

### 4.2. Results

#### 4.2.1. Multi-Target Tracking with the Fixed Clutter Rate

**Figure 3:**
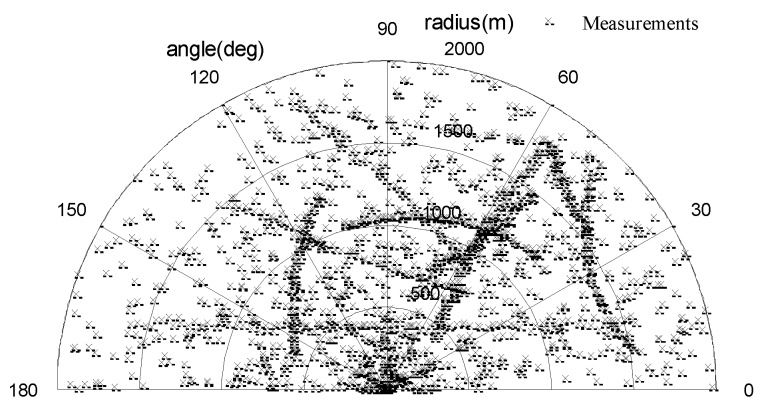
Observations immersed in clutters.

**Figure 4:**
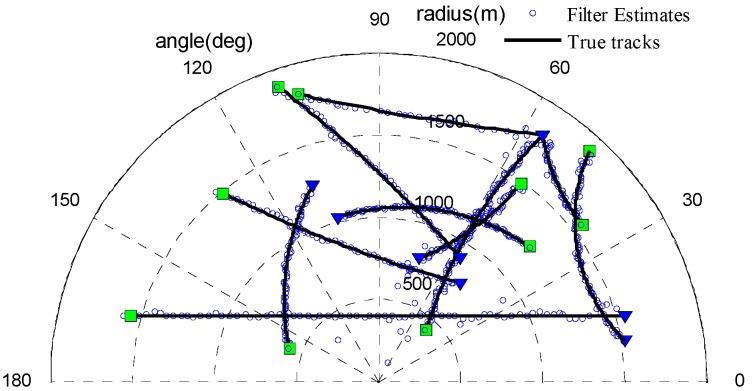
Position estimations of the UCR-MBerF-AI.

In this section, we evaluate the performance of the proposed UCR-MBerF-AI with a fixed clutter rate. There were 100,000 clutter measurements before thresholding. We assume that the measurements can be generated from anywhere within the observation region. The probability of a false alarm is set as $p_{FA,k}^{\tau }=1\times 10^{-4}$, and the corresponding clutter rate after thresholding is λ=10. The SNR of all targets was fixed at 13 dB. According to Equation (4), the probability of detecting actual targets in this case is $p_{D,k}^{\tau }=0.98$. Other scenario parameters are given in Section 4.1. Figure 3 shows the observations immersed in the clutters, while Figure 4 shows the estimated position output of the UCR-MBerF-AI from a single run. Compared with the true trajectories, the results indicate that the UCR-MBerF-AI can correctly determine actual target appearance, motion and disappearance, and achieve accurate multi-target tracking without the need of the new-born objects’ prior RFSs. Moreover, it should be noted that, although false estimates occur occasionally, due to the fact that new-born objects are driven by the measurements, the false estimates die out very quickly.

**Figure 5:**
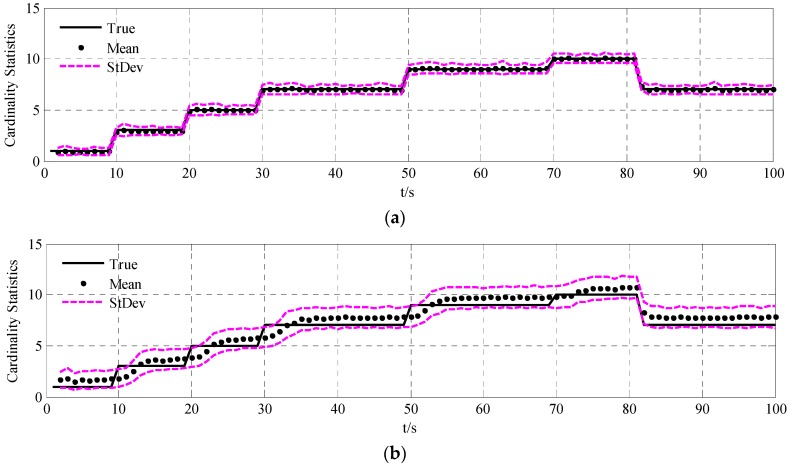
Cardinality statistics for the fixed clutter rate scenario. (**a**) UCR-MBerF-AI; (**b**) UCR-MBerF.

**Figure 6:**
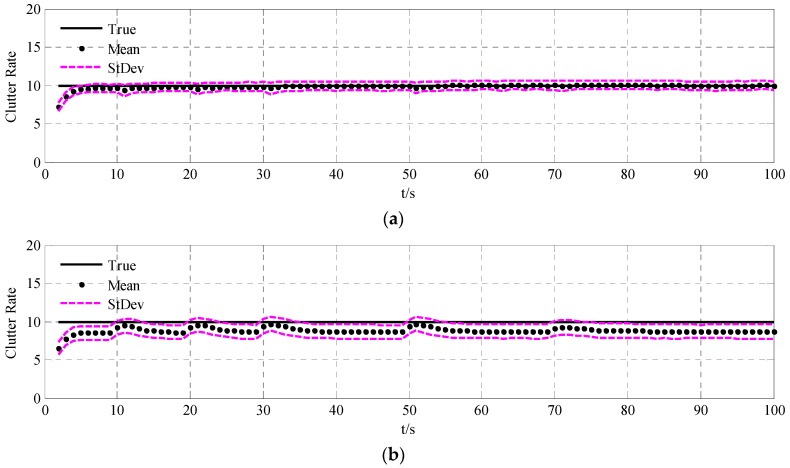
True and estimated clutter rates for the fixed clutter rate scenario. (**a**) UCR-MBerF-AI; (**b**) UCR-MBerF.

To validate the performance of the UCR-MBerF-AI, it was compared to that of the UCR-MBerF [22] over 100 Monte Carlo (MC) trials. The target trajectories shown Figure 2, along with random clutters were used for each trial. Figure 5 and Figure 6 show the cardinality and clutter rate statistics *versus* time for the UCR-MBerF-AI and the UCR-MBerF, respectively. These results confirm that the UCR-MBerF-AI produces more accurate estimations of both the number of actual targets and the clutter rate than the UCR-MBerF. In addition, the UCR-MBerF has a negative bias in the clutter rate estimate and a corresponding positive bias in the cardinality estimate. This is because some clutters are treated as actual targets, while the UCR-MBerF-AI uses amplitude information to distinguish between actual targets and clutters.

We assessed the performance of the given multi-target filters using the OSPA metric, which can jointly evaluate the localization and cardinality error between two finite sets [25]. The OSPA metric is given by Equation (28) with p=1, c=300:

(28) $${\bar{d}}_{p}^{\left(c\right)}(X,Z)={\left(\frac{1}{n}\left(\underset{\pi \in \prod_{n}}{\min}\sum_{i=1}^{m}d^{\left(c\right)}{\left(x_{i},z_{\pi \left(i\right)}\right)}^{p}+c^{p}(n-m)\right)\right)}^{\frac{1}{p}}$$

**Figure 7:**
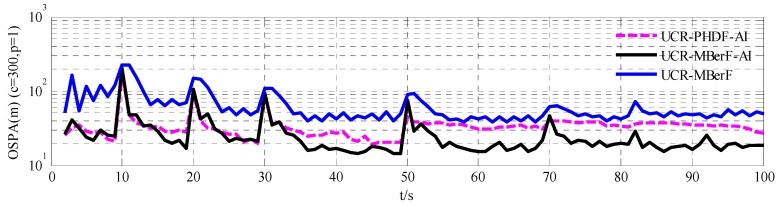
OSPA metrics of UCR-PHDF-AI, UCR-MBerF-AI and UCR-MBerF for the fixed clutter rate scenario.

We compared the UCR-MBerF-AI with the UCR-PHDF-AI and the UCR-MBerF. Figure 7 shows the mean estimation errors obtained using the OSPA metric for the 100 MC simulations over time. It can be seen that the performance of the UCR-MBerF-AI is better than the UCR-PHDF-AI, while the UCR-MBerF performs significantly worst among the three filters. The main reason is that the UCR-MBerF-AI performs more accurate estimations and obtains a smaller variance of the cardinality than the UCR-MBerF, as shown in Figure 5, while being free of the reliance on clustering that is a necessary step in the implementation of the UCR-PHDF-AI. Due to the change of the cardinality, both the UCR-MBerF-AI and the UCR-MBerF produce significant peaks at 10, 20, 30, 50, 70 and 82 s, however the UCR-PHDF-AI only produces significant peaks at 10, 20 and 30 s. This is because there are more targets in the second half of the simulation, and the UCR-PHDF-AI performs worse due to the clustering process.

#### 4.2.2. Multi-Target Tracking with the Time-Varying Clutter Rate

**Figure 8:**
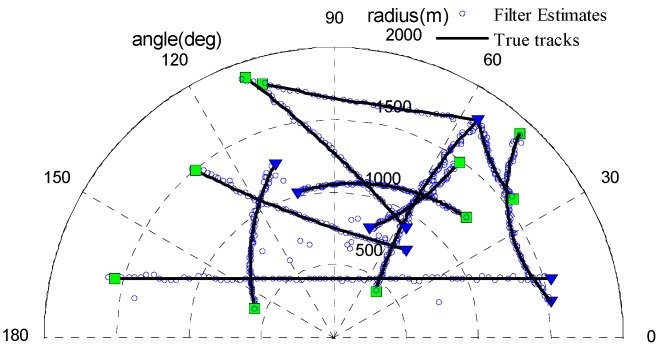
Position estimations of the UCR-MBerF-AI.

To demonstrate the performance of the UCR-MBerF-AI in clutter rate estimation, we consider the scenario with the target trajectories shown in Figure 2 and a time-varying clutter rate. The scenario parameters are as same as in Section 4.1, except for the number of the clutters before thresholding. Figure 8 shows the UCR-MBerF-AI output of the estimated target positions from a single run.

**Figure 9:**
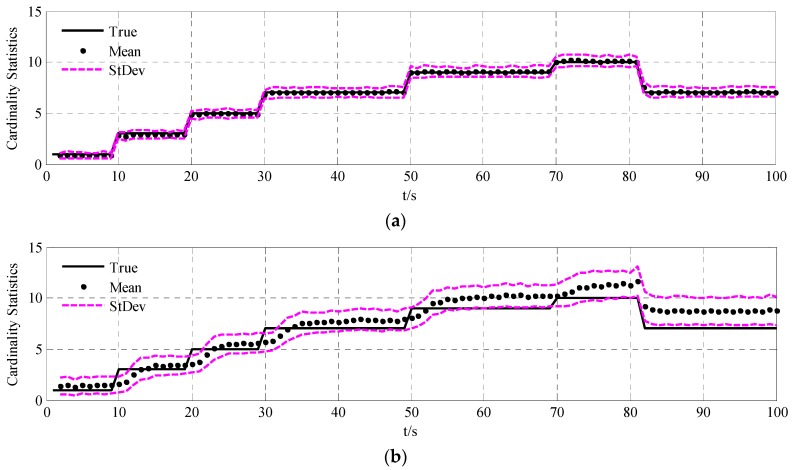
Cardinality statistics for the time-varying clutter rate scenario. (**a**) UCR-MBerF-AI; (**b**) UCR-MBerF.

**Figure 10:**
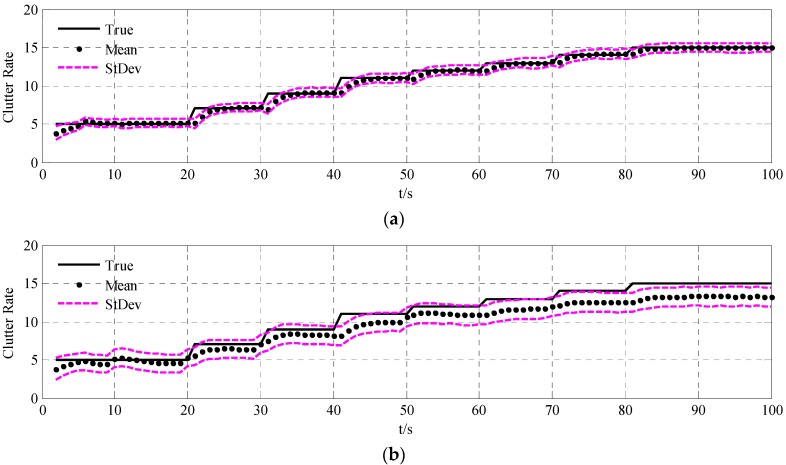
True and estimated clutter rates for the time-varying clutter rates scenario. (**a**) UCR-MBerF-AI; (**b**) UCR-MBerF.

The averaged cardinality and clutter rate statistics *versus* time for the 100 MC simulations using the UCR-MBerF-AI and UCR-MBerF are shown in Figure 9 and Figure 10, respectively. It can be seen that UCR-MBerF-AI can estimate the number of actual targets accurately, whereas the UCR-MBerF has a positive bias which affects its performance adversely as the clutter rate increases. Moreover, the UCR-MBerF-AI provides a more accurate estimation in the clutter rate, while the UCR-MBerF shows a comparatively accurate estimation when the clutter rate is below 5 but demonstrates a negative bias when the clutter rate is larger; this bias becomes more noticeable as the clutter rate increases.

**Figure 11:**
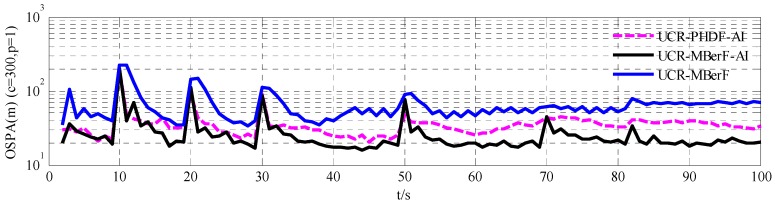
OSPA metrics of the UCR-PHDF-AI, UCR-MBerF-AI and UCR-MBerF for the time-varying clutter rates scenario.

Figure 11 shows the mean estimation errors obtained using the OSPA metric for the 100 MC simulations over time. The UCR-MBerF-AI, UCR-PHDF-AI and UCR-MBerF produce approximately average errors of 25, 36 and 50 m per target, respectively. This is due to the more accurate estimation of the number of actual targets and clutter rate of the UCR-MBerF-AI. These experimental results further suggest that the UCR-MBerF-AI outperforms the UCR-PHDF-AI, which in turn outperforms the UCR-MBerF.

#### 4.2.3. Multi-Target Tracking at Various SNR Levels

To discuss the effect of the noise on the UCR-MBerF-AI, 100 MC trials were performed for various SNR values. The same target trajectories shown in Figure 2 with random clutters for each trial were also adopted, with an average of 10 clutters per scan after thresholding. The average OSPA metrics and corresponding estimated clutter rate for the three filters *versus* the SNR are shown in Table 1 and Table 2, respectively. As expected, the average OSPA metrics increase and the accuracy of estimated clutter rate decreases as the SNR decreases for the three filters. This is because the detection probability decreases with the lower SNR, and these filters are all more sensitive to increased noise levels, which make detection more difficult. However, the UCR-MBerF-AI still performs better than the UCR-PHDF-AI and UCR-MBerF, as seen by the lower location and cardinality errors.

**Table 1:** Time-averaged OSPA metrics for various SNR with $p_{FA,k}^{\tau }=1\;\times \;10^{-4}$.

SNR	10.50 dB	11.12 dB	11.85 dB	13.00 dB	14.50 dB
$p_{D,k}^{\tau }$	0.70	0.80	0.90	0.95	0.99
UCR-MBerF-AI	102.63	74.72	49.25	26.39	24.85
UCR-PHDF-AI	116.54	91.83	64.61	37.72	34.95
UCR-MBerF	129.88	107.48	87.98	63.47	57.84

**Table 2:** Time-averaged estimated clutter rate for various SNR with $p_{FA,k}^{\tau }=1\;\times \;10^{-4}$.

SNR	10.50 dB	11.12 dB	11.85 dB	13.00 dB	14.50 dB
$p_{D,k}^{\tau }$	0.70	0.80	0.90	0.95	0.99
UCR-MBerF-AI	9.65	9.72	9.85	9.94	9.98
UCR-PHDF-AI	9.03	9.17	9.26	9.35	9.53
UCR-MBerF	8.56	8.73	8.90	9.15	9.32

## 5. Conclusions

In this paper, we have presented an improved MBerF, named the UCR-MBerF-AI, which not only achieves more accurate and steady estimations of the clutter rate and the number of actual targets, but also relaxes the requirement on the *a priori* knowledge of the new-born object RFSs. The proposed improved filter retains the mathematical structure of the conventional MBerF, while additionally incorporating the signal amplitude information into the MBerF recursion loop to enhance the discrimination between actual targets and clutters, and utilizing measurements to adaptively generate the new-born object RFSs. Moreover, the SMC implementation of the proposed filter was studied using numerical simulations. The results demonstrate that the UCR-MBerF-AI improves the estimation accuracy of the target number and the corresponding multi-target states, as well as the clutter rate, over previous approaches.
